# Controversial Character of This No. of the Journal

**Published:** 1857-05

**Authors:** 


☞ We wish it distinctly understood, that the apparently controversial character of this number of our Journal, is entirely the result of a conviction, upon our part, that a regularly concocted plan has been formed for our injury: this we state, as our belief, from what we consider almost positive testimony, and though, what we have said, is certainly defensive, it would not have been said, but for the belief that "forbearance had ceased to be a virtue."

Our efforts in the cause of medical science, though feeble, have proceeded from an honest desire to advance it in every particular, and in reference to the subject of medical education, we have long since announced ourselves as ready to come up to the aid of the profession in the elevation of the standard, and not having violated any principle, we have not felt it to be our duty to submit in silence to the unjust aspersions which have been cast upon us.

In reference to this subject, more than a year since, in the March number of this Journal, for 1856, we held the following language, which we now repeat:

"The projectors of this enterprise are not under the influence of any such selfish and contracted view, as to have for their object, merely to build up a patronage, and to vie with rival schools for *numbers*, but with a comprehensive sense of the obligations resting upon them, as the representatives of a science, devoted to the elucidation of the most important truths connected with our temporal existence, are laboring to lay a broad and solid foundation, with an enduring superstructure, that will, in a higher and more important sense, compete successfully with the first institutions in the land; and if there are any individuals or institutions belonging to the profession, whose course indicates, that in their estimation, "they are the people, and wisdom will die with them"—and who are setting themselves up as the general regulators of medical affairs, and are disposed to dictate what we shall, or shall not, do, we would say to them, that while we are perfectly satisfied with our position, (and the ample recognition of our claims, upon the part of other medical colleges,) as being fully endowed with rights and powers, fully equal to those emanating from any government in this or any other country, and therefore, if preferred, perfectly independent of any *self-constituted* tribunal whatever, we still choose to place ourselves upon the same platform with the other representatives of true and regular medicine, and wish it to be distinctly understood, that our intention is to have a standard not inferior, in any particular, to that which shall be adopted and sustained by the voice and action of the profession, and in whatever matter, reform or improvement, may be demanded, we shall be prepared to come up to the full requisition, and shall, at all times be found as active and zealous coöperators for the more thorough education of medical men —whether it be by more rigid requirements in reference to preliminary education, a higher standard to regulate examinations, the separation of the teaching and licensing power, or a prolonged term of study; and in reference to the latter, we do not mean those catchpenny and pseudo extensions of terms, denominated preliminary lectures, whose object is now too well understood to make it necessary to do more than barely allude to them, but a *bona fide* establishment of some uniform increase in the time of study and attendance upon lectures, before an individual shall be allowed to become a candidate for the degree."

We earnestly hope that this will be the end of it, and that we may be permitted to go on in peace, and be tested by our merits; but let the consequences be what they may, we shall continue to speak the truth without fear.

